# Supplementary material for: MetaRibo-Seq measures translation in microbiomes
Source: Nat Commun. 2020 Jun 29;11:3268. doi: 10.1038/s41467-020-17081-z (PMC7324362; doi:10.1038/s41467-020-17081-z)
Supplement: Supplementary file 10 — Supplementary Data 7 [file 41467_2020_17081_MOESM10_ESM.zip › File2/Confidence_High_Taxonomy/242463_out.krona.html]

Javascript must be enabled to view this page.

members
magnitude
magnitudeUnassigned
count
unassigned
taxon
rank

242463\_out

6

2
1
superkingdom

phylum
1
201174

1497346
1
class

order
1
588673

361606
family
1

1
genus
361607


SRS1041145\_contig\_number\_19340
298163
species
1

5

SRS013687\_contig\_number\_contig-100\_12242.12242SRS023914\_contig\_number\_26622SRS051031\_contig\_number\_10094SRS053356\_contig\_number\_contig-100\_3946.230142SRS076929\_contig\_number\_14365
